# Electrochemical Analysis of Sulfisoxazole Using Glassy Carbon Electrode (GCE) and MWCNTs/Rare Earth Oxide (CeO_2_ and Yb_2_O_3_) Modified-GCE Sensors

**DOI:** 10.3390/molecules27062033

**Published:** 2022-03-21

**Authors:** Marwa El-Azazy, Insharah Ahsan, Nasr Bensalah

**Affiliations:** Department of Chemistry and Earth Sciences, College of Arts and Sciences, Qatar University, Doha 2713, Qatar; ia1803723@student.qu.edu.qa

**Keywords:** electroanalysis, modified electrode, rare earth metal oxides (REMO), multiwalled carbon nanotubes (MWCNTs), sulfisoxazole, peak current, limit of detection

## Abstract

In this work, new electrochemical sensors based on the modification of glassy carbon electrode (GCE) with multiwalled carbon nanotubes (MWCNTs)—rare metal oxides (REMO) nanocomposites were fabricated by drop-to-drop method of MWCNTs-REMO dispersion in ethanol. REMO nanoparticles were synthesized by precipitation followed by hydrothermal treatment at 180 °C in absence and presence of Triton^TM^ X-100 surfactant. Cyclic voltammetry (CV) analysis using MWCNTs-CeO_2_@GCE and MWCNTs-Yb_2_O_3_@GCE sensors were used for the analysis of sulfisoxazole (SFX) drug in water samples. The results of CV analysis showed that MWCNTs-REMO@GCE sensors have up to 40-fold higher sensitivity with CeO_2_ compared to the bare GCE sensor. The estimated values of the limit of detection (LoD) of this electrochemical sensing using MWCNTs-CeO_2_@GCE and MWCNTs-Yb_2_O_3_@GCE electrodes reached 0.4 and 0.7 μM SFX in phosphate buffer pH = 7, respectively. These findings indicate that MWCNTs-REMO@GCE electrodes are potential sensors for analysis of sulfonamide drugs in water and biological samples.

## 1. Introduction

Sulfonamides (SAs) represent an enormous class of compounds with a variety of pharmacological actions. Being the first recognized broadly effective antibacterials to be used systemically, SAs have paved the way for the antibiotic revolution since 1940. As antibiotics, SAs have played a crucial role as efficient therapeutics both in the human and veterinary rehearsals [1,2]. In the European Union (EU), SAs are the second most consumed category of antibiotics following tetracyclines [3,4]. The extensive use of SAs due to their availability at almost no cost, broad spectrum, and effectiveness even for the nonantibiotic purposes is raising several concerns.

Like the other pharmaceuticals and personal care products (PPCPs), SAs have been widely detected in various aquatic environments [5,6,7,8,9]. Occurrence of SAs in water does not only negatively affect the human and the animal health but might result in the upsurge of antibiotic-resistant bacteria on the long run. Similar situation is encountered for the dairy, meat and poultry products obtained from animals treated with SAs. The EU regulations specifies 100 µg/L as the maximum residue limit of SAs in milk [10].

**Scheme 1:**
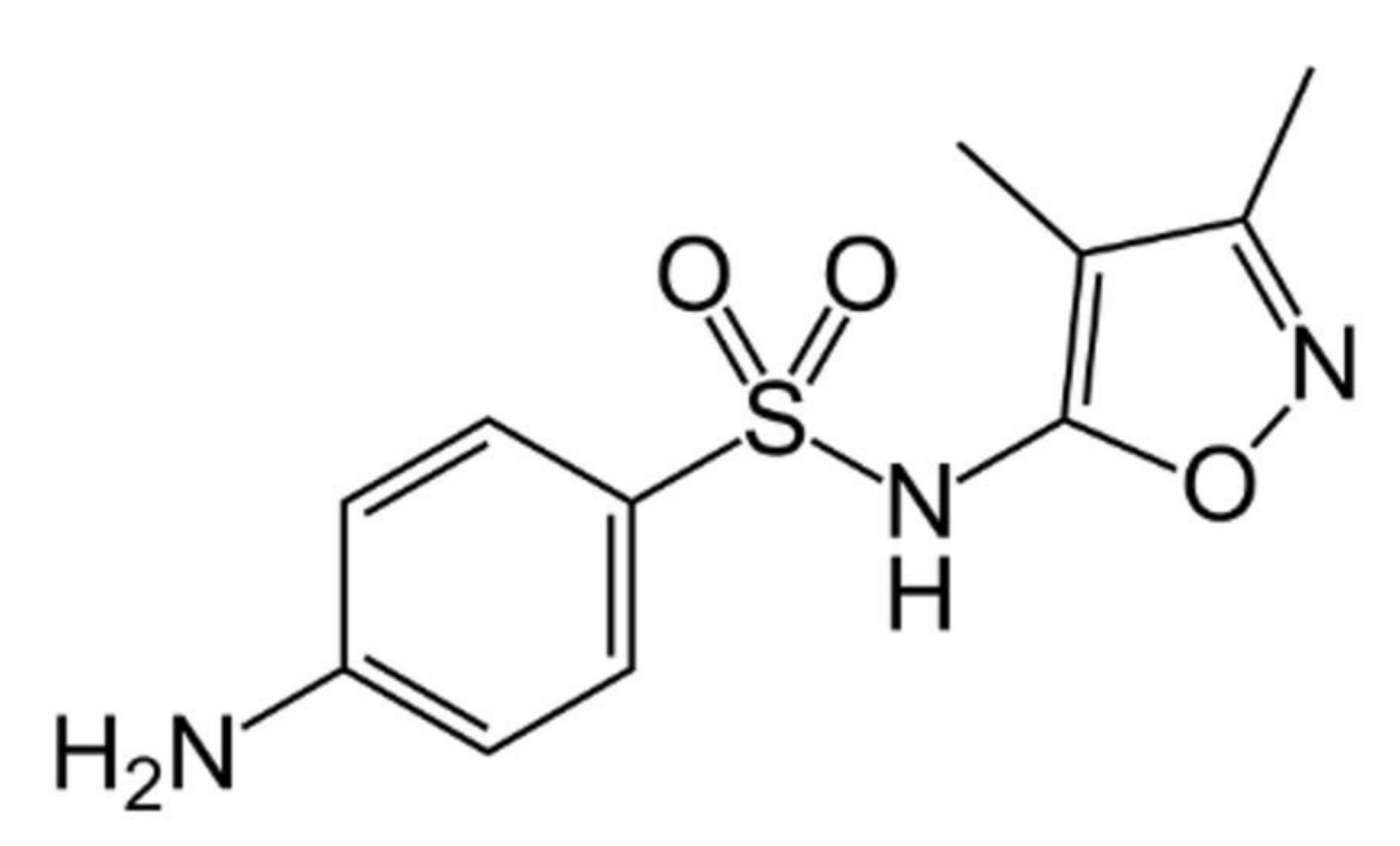
Chemical structure of SFX. Chemical Formula: C_11_H_13_N_3_O_3_S (267.31 g/mol); IUPAC Name: 4-amino-N-(3,4-dimethyl-1,2-oxazol-5-yl)benzenesulfonamide; pK_a1_: 1.5, pK_a2_: 5.0 [12].

Sulfisoxazole (SFX, Scheme 1), a member of the SAs family is an antibiotic that is commonly used for veterinary purposes. SFX is used mainly to treat the bacterial infections in the digestive system and the respiratory tract and as a growth promoter for the livestock [10,11]. Unfortunately, the aftermath of the utilization of SFX for the veterinary purposes has resulted in the evolution of SFX-resistant *E. coli*. Moreover, SFX and since 2017 has been classified as a Group 3 carcinogen [12,13]. Moreover, endocrine disorders and hypersensitivity reactions are among the side effects associated with SFX [14]. Therefore, having an accurate, sensitive, and reliable method for the determination of SFX is crucial.

The complexity of the matrices whether environmental or food products and the occurrence of SFX as traces makes the detection of SFX a challenge. A literature survey shows several procedures for the determination of SFX. Reported efforts included the development of nanohybrid magnetic optosensing probes [14], high performance liquid chromatography (HPLC) [15,16,17], capillary electrophoresis (CE) [18], fluorescence spectroscopy [19], colorimetry [20], and the development of a voltammetric sensor [21]. Each of these approaches has its pros and cons. HPLC for example needs well-trained personnel, expensive, and cannot be afforded in the majority of the quality control laboratories. Colorimetry is a traditional approach that is neither sensitive nor selective. Electrochemical sensing, however, involves simple procedure and setup, real time analysis, better sensitivity, suitable for trace analysis, and does not require expensive instrumentation. Moreover, electrochemical sensing-based approaches are sensitive with a broad linear response, and better reproducibility [22,23,24].

Utilization of chemically modified electrodes helps improving the sensing process. From the one hand, chemical modification serves to minimize the high over-potential needed for the oxidation of organic analytes. From the other hand, it plays a role in reducing the effect of interferents. Recently, hybrid nanostructured materials have been widely employed in sensing applications thanks to their high surface area, reasonable biocompatibility, catalytic activity, chemical stability, and high electrical conductivity [25,26,27,28,29]. Carbon nanotubes (CNTs) and compared to the other carbon electrodes are found to be advantageous in terms of sensitivity, selectivity, response time, reusability, and the resistance to surface fouling [30,31]. Therefore, electrode modification using nanoparticles of rare earth metal oxides (REMO), together with other nanostructures such as multi-wall carbon nanotubes (MWCNTs) could create an ideal sensing interface.

In the current investigation, a glassy carbon electrode (GCE) has been modified utilizing a composite nanomaterial made of the nanoparticles of oxides of both ytterbium and cerium along with MWCNTs in a Nafion binder. The objective is to develop a sensitive, selective, stable, and reproducible electrochemical sensor for the determination of SFX. The developed sensor and to the best of our knowledge is the first voltammetric sensor for SFX utilizing a REMO with MWCNTs in presence of Triton^TM^ X-100 as a surfactant. The constituents of the composite material were successfully characterized. The electrochemical parameters such as pH, scan rates, and concentration have been optimized.

## 2. Materials and Methods

### 2.1. Chemicals and Materials

Sulfisoxazole (SFX) was a product of Biosynth^®^ Carbosynth Ltd. (Compton, Berkshire, UK, >99.9%) and was used as received. Rare earth metal (REM) hydrated nitrates were received from VWR (VWR International Global Exports, Arlington’s Heights, IL, USA) in a high purity grade (>99.0%). Triton^TM^ X-100, MWCNTs, absolute ethanol, and the components of phosphate, oxalate, and borate buffers were analytical grade Sigma–Aldrich chemicals (Sigma–Aldrich, St. Louis, MO, USA). Nafion was a product of Fluka Chemie GmbH (Buchs, Switzerland). The working disk electrodes, the reference electrode, Ag/AgCl (3 M KCl), with a flexible connector, and the platinum wire auxiliary electrode (6.5 cm) with gold-plated connector were purchased from Bioanalytical Systems, Inc. (BASi^®^, West Lafayette, IN, USA). Disk electrodes were manufactured in a solvent-resistant PCTFE plastic body (7.5 cm length × 6 mm OD) which is embedded with highly polished disks of glassy carbon electrodes (GCE, MF-2012, 3.0 mm diameter).

### 2.2. Preparation of Rare Metal Earth Oxide Nanoparticles

Pure rare earth metal oxides (REMO) nanoparticles (CeO_2_ and Yb_2_O_3_) were prepared in three stages: precipitation, hydrothermal treatment, and annealing. Cerium (III) nitrate hexahydrate, Ce(NO_3_)_3_·6H_2_O, and ytterbium (III) nitrate pentahydrate, Yb(NO_3_)_3_·5H_2_O were dissolved in 50 mL of deionized water. The complete dissolution of the REM nitrates was ensured by magnetic stirring at 400 rpm during 1 h at 60–70 °C. A little bit excess to the stoichiometric amount of sodium hydroxide (NaOH) was then added to the dissolved REM nitrates aqueous solutions to precipitate the REM hydroxides (Ce(OH)_3_ and Yb(OH)_3_). The solutions containing the suspended solids were transferred into Teflon jars/liners, and then jars were introduced into autoclaves (stainless steel body) and were tightly sealed. The autoclaves were inserted in the oven where they were hydrothermally treated at 220 °C for 24 h. The precipitated solids obtained after the hydrothermal treatment were separated by centrifugation at 6000 rpm during 10 min. The solids were washed several times with water until a neutral pH was maintained and then by ethanol. The separated solids were dried at 80 °C in a vacuum oven overnight. The dried solids were heat-treated under atmospheric air in a tube furnace at 550 °C for 6 h. The weights of the samples were measured before and after heat treatment to ensure the dehydration of the solids obtained at the end of the synthesis. The final products were stored in Teflon vials for further utilization. In order to boost the morphology, decrease the particle size and to get more uniform shapes of REMO nanoparticles, the same stages were performed in presence of a surfactant (Triton X-100). Porous and uniform metal oxides were prepared in presence of Triton X-100.

### 2.3. Fabrication of MWCNTs/CeO_2_ and MWCNTs/Yb_2_O_3_ Modified-GCE

Desired amounts of the as-prepared CeO_2_ and Yb_2_O_3_ were added to an ethanolic suspension of MWCNTs containing 2 mg/mL MWCNTs at different (REMO/MWCNTs) mass ratios (1:4, 1:2, 1:1, 2:1). The MWCNTs/REMO mixed suspension was sonicated in ultrasound water bath for 30 min at 20–25 °C in presence of 20 μL of 5% Nafion solution in ethanol to ensure the coating of MWCNTs by the REMO nanoparticles. Aliquots of 5–20 μL of MWCNTs/REMO suspension were cast over the surface of polished GCE electrode by drop-to-drop method. The modified-GCE was dried in the oven at 80 °C for 15 min to evaporate the solvent and maintain the MWCNTs/REMO thin film on the top of GCE surface with the help of Nafion polymer used as binder. The dried MWCNTs/REMO/GCE sensor was washed several times by immersion in phosphate buffer solution before use.

### 2.4. Characterizations of REMO Nanoparticles and MWCNT/REMO Composites

The crystalline structure of REMO powders was determined using the X-ray diffraction spectroscopy (PAnalytical Empyrean X-ray diffractometer, 40 KV/30 mA, Cu-K_α_ radiation) at a scan rate of 2°/min between 20° and 90°. The morphology of the REMO nanoparticles and the particle size observation was conducted by FEI NOVA NANOSEM 450 scanning electron microscope (SEM). Energy dispersive X-ray (SEM-EDX) analysis was performed to determine the elemental composition of REMO solids. The MWCNT/REMO composites were observed using transmission electron microscopy (TEM, TEM JEOL JEM-2100F) operated at 200 kV.

### 2.5. Preparation of the SFX Standard Solutions

All the preparations were performed in buffered aqueous solutions prepared by dissolution of the buffer system in deionized water received from the Mill-Q^TM^ system with a resistivity ≥18 MΩ cm^−1^. A stock solution of SFX (100 μM) was prepared in different buffer aqueous solutions (oxalate buffer: pH = 4.0, phosphate buffer: pH = 7.0, and borate buffer: pH = 9.0). Serial dilutions of SFX (0.1 to 200 μM) were prepared in the same solvent.

### 2.6. Electrochemical Measurements

Cyclic voltammetry (CV) and square wave voltammetry (SWV) were performed in three electrode electrochemical cell using GCE and modified-GCE as working electrode, platinum (Pt) wire as counter electrode, and Ag/AgCl as reference electrode in different buffered media. The effects of the scan rate, the pH of the buffer solution, the composition of the modified-GCE, and the concentration of the analyte on the electrochemical behavior of SFX were evaluated. The changes of the peak currents with the scan rate, buffer pH, and concentrations were plotted for SFX using the GCE and modified-GCE electrochemical sensors.

## 3. Results and Discussion

### 3.1. Characterizations of REMO Solids and MWCNTs/REMO Composites

By and large, the physical and chemical properties as well as the performance of nanomaterials depend not only on their composition but also on their particle size, and the particle size distribution. Controlling the variables affecting the synthetic process helps having a control over the particle size and the particle size distribution [32,33,34]. The effect of the presence of surfactants during the hydrothermal synthesis of metal oxides have been reported in literature [33,34,35,36,37,38,39,40,41]. In general, surfactants are known for their ability to exert a control over the morphology and the crystal growth of inorganic nanoparticles, most likely because of the adsorption of the surfactant on the surface of the nanoparticles hindering their further growth. In the current investigation, a nonionic surfactant, Triton^TM^ X-100, was employed to have a control on the particle size of REMO nanoparticles. A comparison of the morphological properties in presence and absence of surfactants was held.

**Figure 1:**
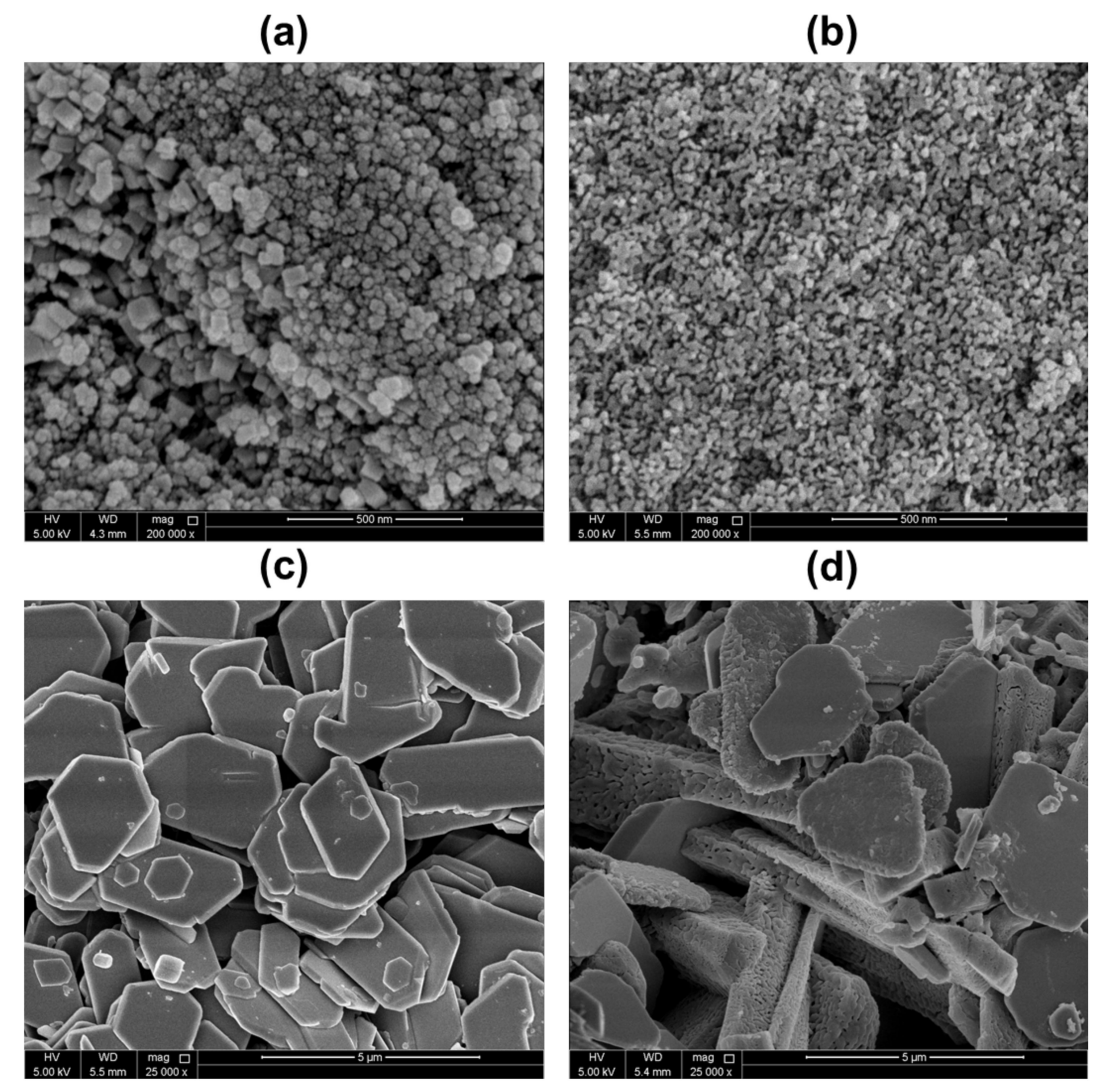
SEM images of CeO_2_ (**a**,**b**) and Yb_2_O_3_ (**c**,**d**) synthesized by hydrothermal method followed by annealing in absence (**a**,**c**) and in presence (**b**,**d**) of Triton^TM^ X-100 as a surfactant.

Figure 1 presents the SEM micrographs of CeO_2_ (a and b) and Yb_2_O_3_ (c and d) prepared by the hydrothermal treatment in absence (a and c) and in presence of Triton X-100 (b and d). The morphology of both REMO nanoparticles is affected by the presence of Triton X-100 surfactant. The as-prepared CeO_2_ nanoparticles presented an inhomogeneous morphology with the appearance of agglomerated particles with cubic and spherical shapes (50–100 nm) in absence of the surfactant (Figure 1a). The addition of Triton X-100 surfactant during the synthesis of CeO_2_, however, has resulted in an improvement of the morphology of the CeO_2_ nanoparticles and a net decrease in the particle size (10–20 nm) (Figure 1b). The SEM micrographs in Figure 1c show that in absence of the surfactant, compact Yb_2_O_3_ flat sheets with dimensions in the range between 1–133 μm and a thickness between 200–500 nm were formed. However, the porous Yb_2_O_3_ flat sheets were observed in presence of Triton X-100 with similar dimensions and thickness than in absence of the surfactant (Figure 1d).

**Figure 2:**
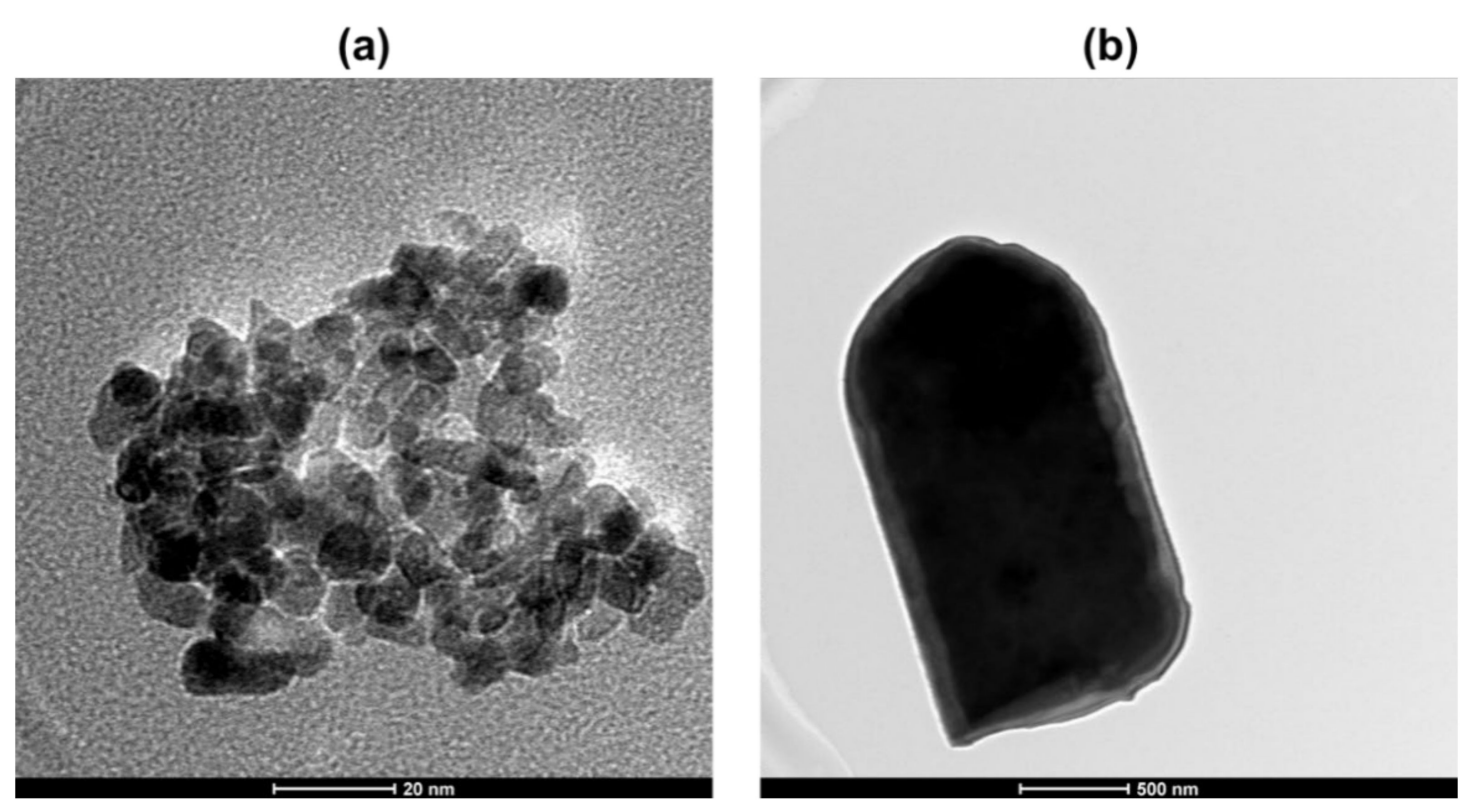
TEM images of (**a**) CeO_2_ and (**b**) Yb_2_O_3_ synthesized by hydrothermal method followed by annealing in presence of Triton^TM^ X-100 surfactant.

The TEM images of CeO_2_ and Yb_2_O_3_ synthesized by the hydrothermal method followed by annealing in presence of Triton^TM^ X-100 surfactant are given in Figure 2. Shown micrographs confirm the results of SEM analysis about the shape and the size of particles of both REMO nanoparticles.

**Figure 3:**
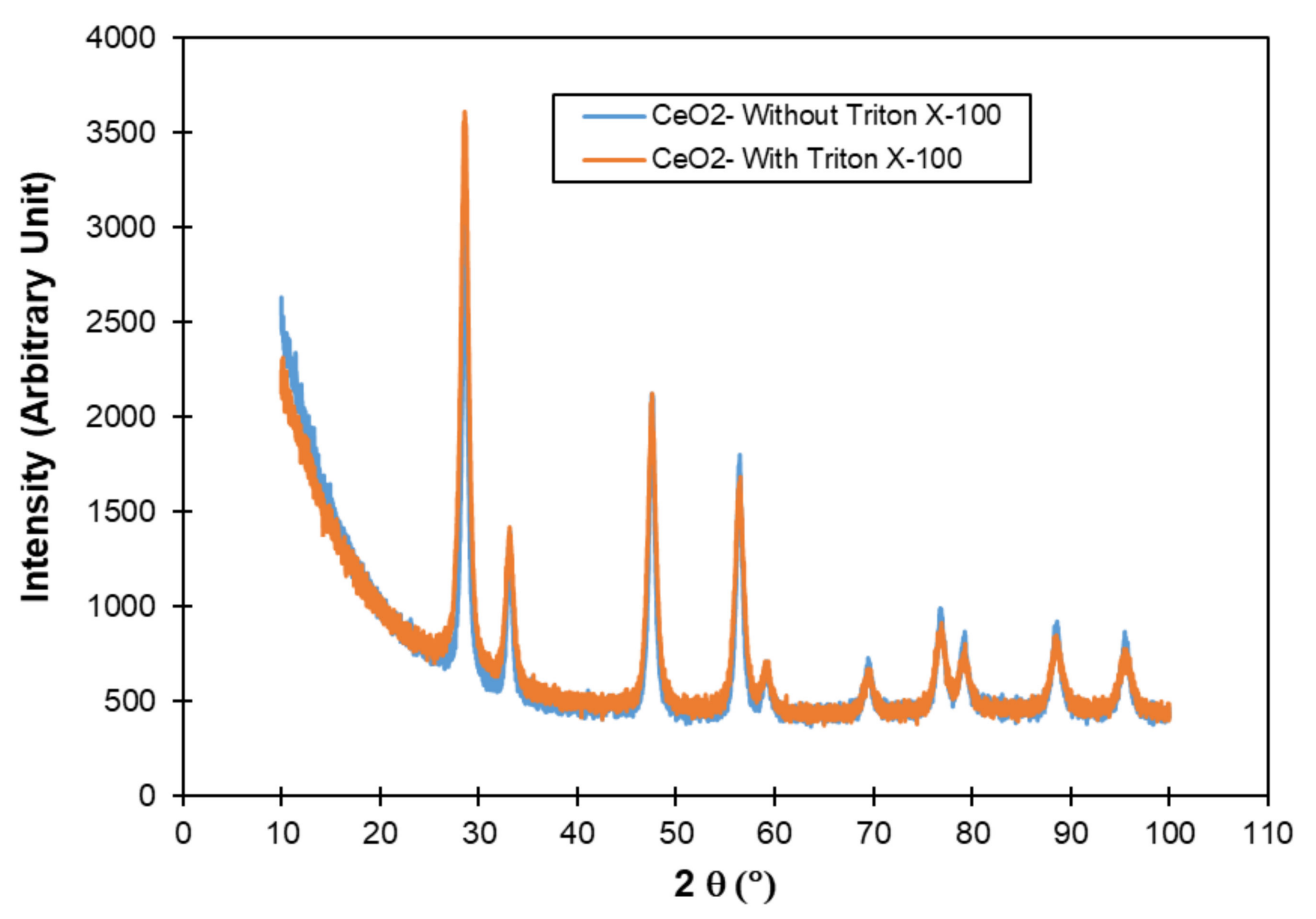
XRD spectra CeO_2_ synthesized by hydrothermal method followed by annealing in absence and presence of Triton^TM^ X-100 surfactant.

Figure 3 presents the XRD spectra of CeO_2_ powders synthesized by hydrothermal method followed by annealing in absence and presence of Triton^TM^ X-100. As it can been seen, the XRD spectra of CeO_2_ prepared in absence and in presence of Triton X-100 coincide to each other indicating that the crystalline structure is not affected by the addition of surfactant.

The XRD peaks for both compounds are located at 28.6, 33.1, 47.5, 56.4, 59.2, 69.5, 76.8, 79.2, and 88.6°. All these XRD peaks correspond to the cubic CeO_2_ (reference#: JCPD 62-1716). This confirms the formation of CeO_2_ cubic crystalline structure at the end of the synthesis procedures including precipitation, hydrothermal treatment, and annealing in open air. Similar results were obtained for Yb_2_O_3_ and no differences were observed between the XRD of Yb_2_O_3_ synthesized in absence and in presence of Triton^TM^ X-100. All the peaks coincide with cubic Yb_2_O_3_ (reference#: JCPDS #65-3173).

**Figure 4:**
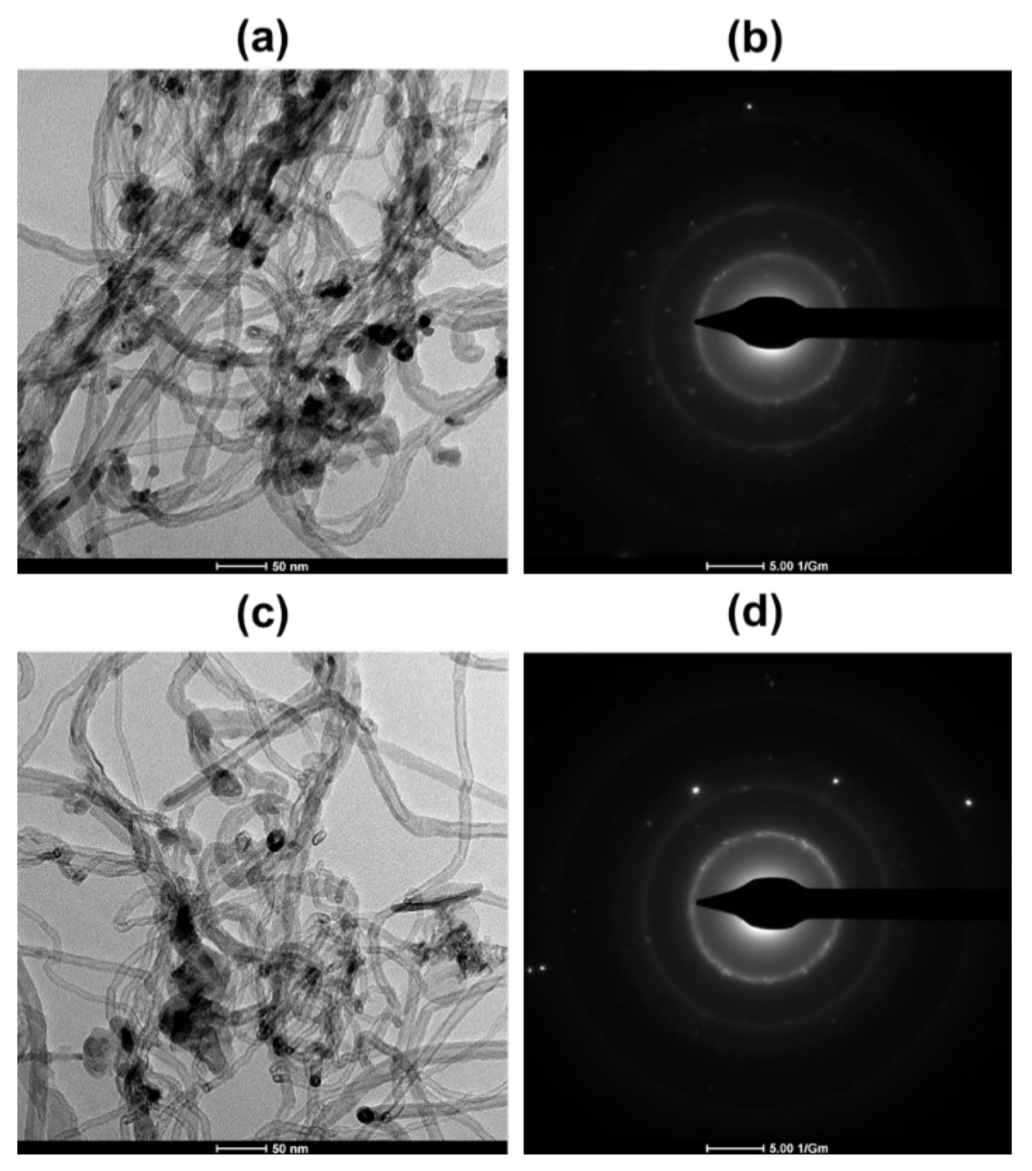
HRTEM images of (**a**) MWCNTs-CeO_2_ and (**b**) MWCNT-Yb_2_O_3_ (1:4 mass ratio) prepared by sonication in presence of Nafion, and SAED patterns (**c**) MWCNTs/CeO_2_ and (**d**) MWCNTs/Yb_2_O_3_.

The as-prepared CeO_2_ and Yb_2_O_3_ were mixed with MWCNTs to prepare the MWCNTs-REMO composites with higher electric conductivity than pure REMO powders. The MWCNTs- REMO composites were prepared in ethanol by sonication of a suspension of 2 mg/mL MWCNT in ethanol with desired amounts of CeO_2_ and Yb_2_O_3_ in presence of Nafion as a binder. Figure 4 presents the TEM images of MWCNTs-CeO_2_ and MWCNTs-Yb_2_O_3_ composite materials. HRTEM shows the decoration and coating of MWCNTs framework with CeO_2_ and Yb_2_O_3_ nanoparticles (see Figure 4a,c). The selected area electron diffraction (XAED) patterns confirm the crystalline structure of CeO_2_ and Yb_2_O_3_ deposited on MWCNTs framework (see Figure 4b,d). The SAED fringes observed in Figure 4b coincide with the diffraction of (111) and (022) planes of CeO_2_.

### 3.2. Analysis of SFX Using Bare GCE Sensor

**Figure 5:**
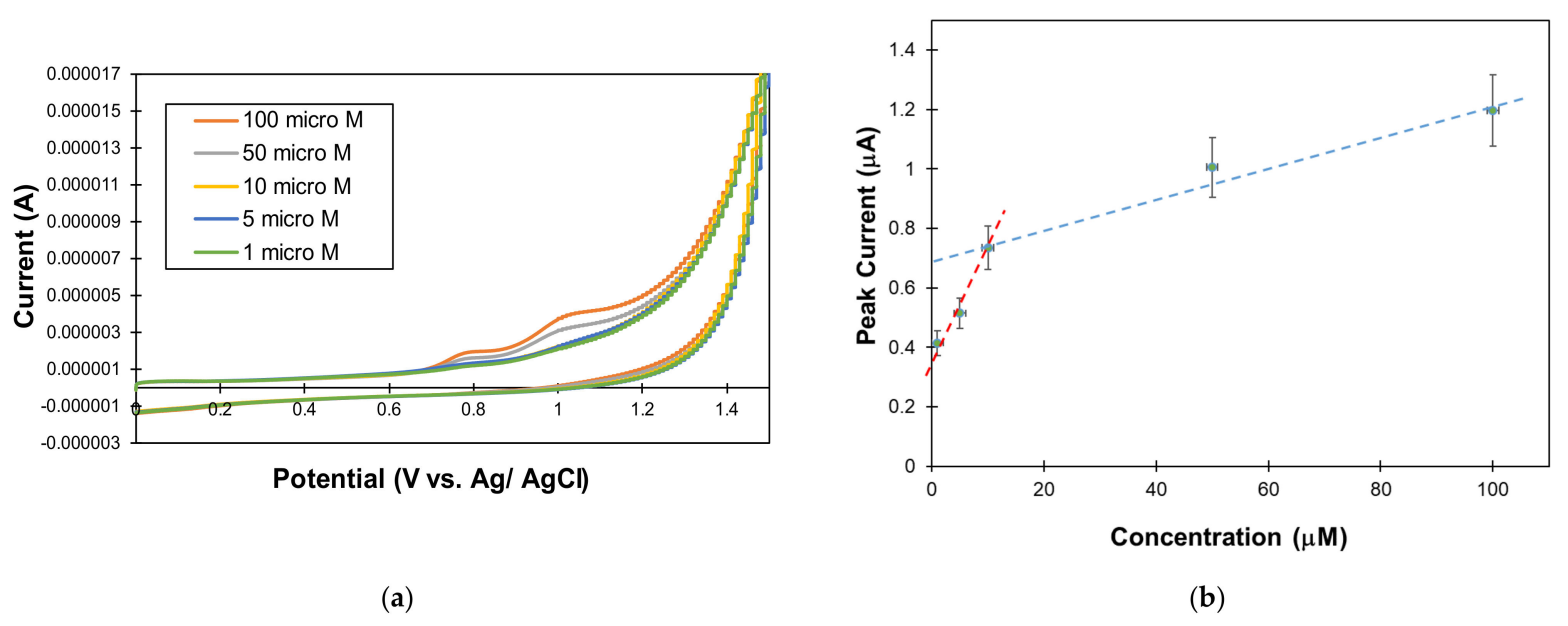
(**a**) Cyclic voltammograms and (**b**) Calibration curve of SFX solutions using GCE electrode in phosphate buffer solution, pH = 7.00 at 50 mV·s^−1^. Experimental conditions: counter electrode: Pt, reference electrode: Ag/AgCl; room temperature; stagnant solution.

The anodic cyclic voltammograms (CV) of SFX at different concentrations in the range of 1.00–100 μM using GCE sensor in phosphate buffer pH = 7.0 at 50 mV·s^−1^ are plotted in Figure 5a. The CV of SFX presents two irreversible anodic peaks at 0.8 and 1.05 V versus Ag/AgCl within the electroactivity window of GCE. The current of both peaks decreased in intensity with the decrease of the SFX concentration indicating that GCE is sensitive detector for SFX. However, the calibration curve plotting the change of the first peak current with SFX concentration in the range of 1.00–100 μM shows two linear areas of dependence: From 0 to 10 μM and from 10 to 100 μM (see Figure 5b). Although GCE electrochemical sensor showed good sensitivity for SFX in the range 1.00–100 μM, it is not a good sensor recommended for concentrations SFX due to the dependence of its sensitivity on SFX concentration.

**Figure 6:**
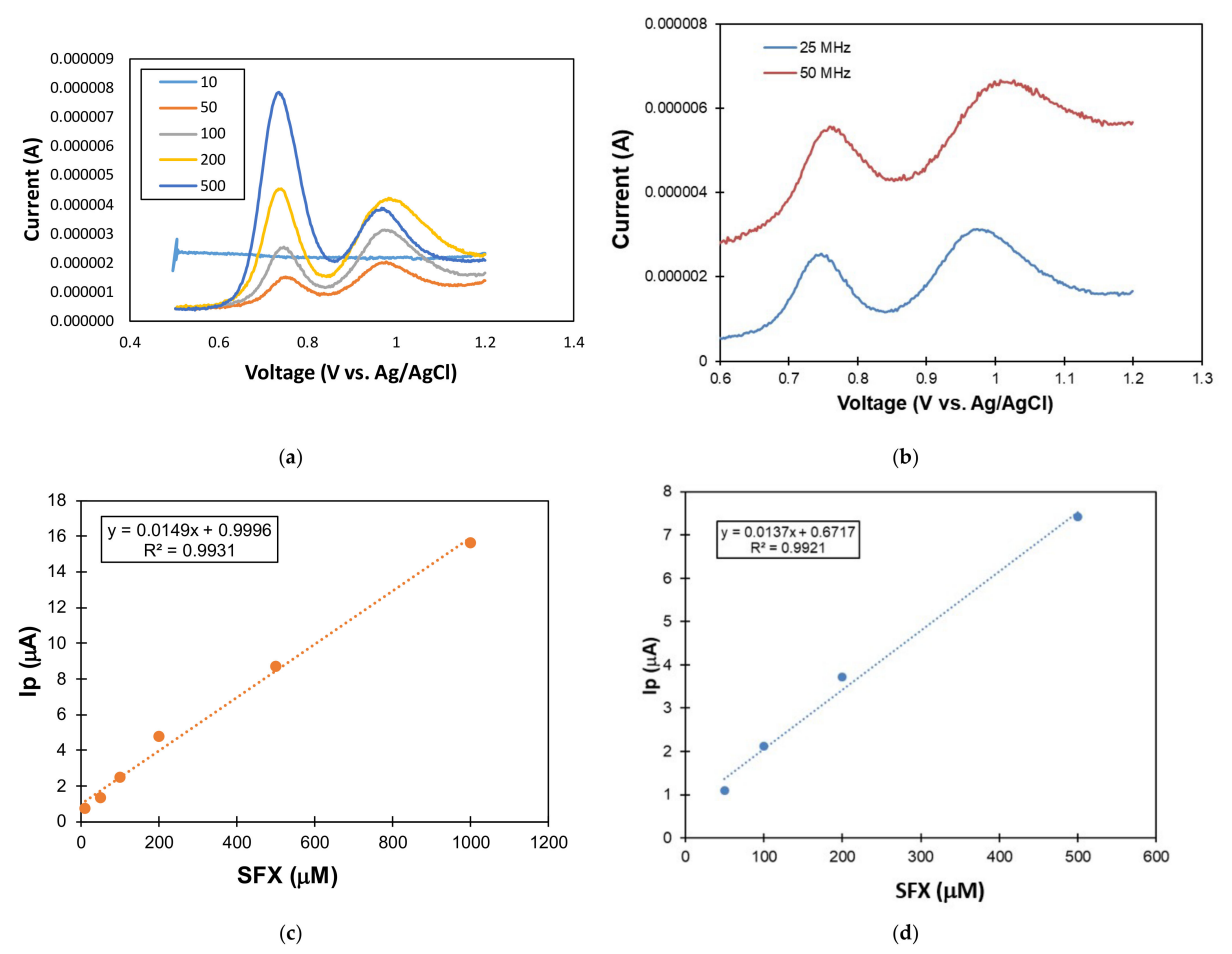
(**a**) Square wave voltammograms of SFX at 25 MHz at different concentrations (10–500 µM), (**b**) Effect of the frequency on the voltammogram of 100 µM SFX. Calibration curves of SFX at: (**c**) 50 MHz and (**d**) at 25 MHz. Experimental conditions: working electrode: GCE (3 mm diameter), counter electrode: Pt wire, reference electrode: Ag/AgCl (3 M KCl), electrolyte: 0.05 M phosphate buffer (pH = 7.0), temperature: 25 °C, stagnant solution.

Figure 6 presents the results of the analysis of SFX by SWV and using a GCE electrochemical sensor in phosphate buffer (pH = 7.0) in the concentration range of 10–1000 μM at two different frequencies 25 and 50 MHz. The SWV plots present two anodic peaks at 0.75 and 0.98 V versus Ag/AgCl at 25 MHz. The current of both peaks increases with the concentration of SFX (Figure 6a) and with the frequency (Figure 6b). The calibration curves related to change of the first peak current (I_p_) with SFX concentration at 50 and 25 MHz are plotted in Figure 6. These graphs show linear profiles for concentrations in the range 10–1000 μM at 50 MHz (Figure 6c) and 50–500 μM for 25 MHz (Figure 6d). This indicates that SWV can reach lower detection limit compared to CV and has better sensitivity for SFX in phosphate buffer. This method, therefore, can be adopted for the electrochemical analysis of SFX using GCE electrode for concentrations higher than 10 μM.

### 3.3. Analysis of SFX Using MWCNTs-REMO Modified GCE Sensors

**Figure 7:**
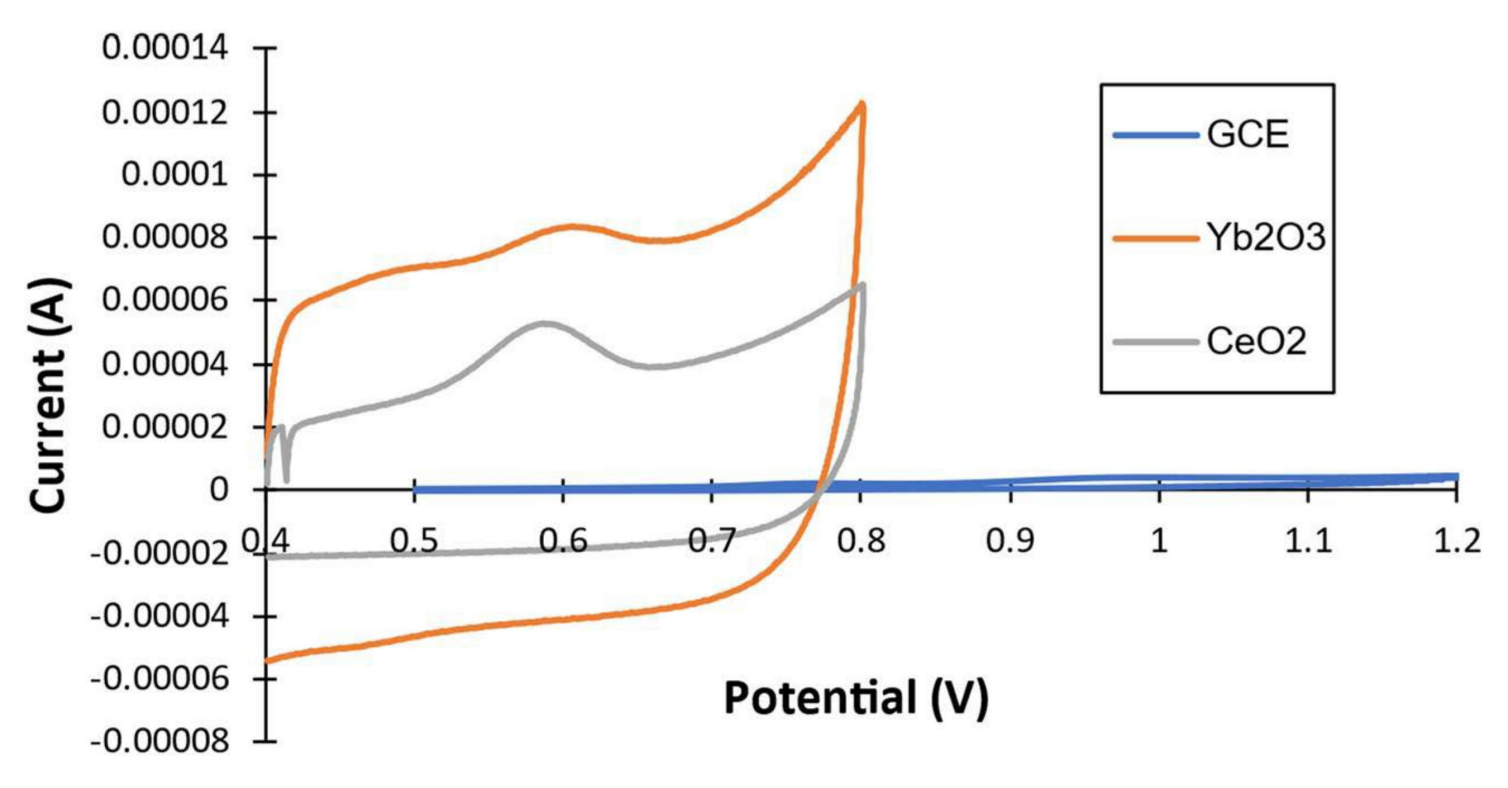
Voltammograms for electrochemical analysis of SFX using GCE electrode, MWCNTs-CeO_2_@GCE electrode and MWCNTs-Yb_2_O_3_@GCE electrode in phosphate buffer solution, pH = 7.0 at 50 mV·s^−1^ for 20 µM. Experimental conditions: counter electrode: Pt, reference electrode: Ag/AgCl; room temperature; stagnant solution.

Figure 7 presents the CVs of 20 μM SFX solution using bare GCE sensor together with MWCNTs-CeO_2_@GCE and MWCNTs-Yb_2_O_3_@GCE as modified GCE sensors in phosphate buffer pH = 7.0 at 50 mV·s^−1^. The modified GCE sensors were prepared by drop-to-drop method of 20 μL of ethanolic suspension containing 2 mg/mL MWCNTs and 0.5 mg/mL CeO_2_ or Yb_2_O_3_ and 10 μL/mL of 5% Nafion solution. As seen, the replacement of GCE with MWCNT-CeO_2_@GCE and MWCNT-Yb_2_O_3_@GCE sensors shifted the first peak to lower voltages and increased its current. The electrochemical oxidation of SFX on the surface of MWCNT-CeO_2_ and MWCNT-Yb_2_O_3_ happens at lower potential due to higher electroactivity of the GCE modified electrodes and the larger surface area through nanostructuring confirmed by the increase of the current peak. The peak current increased from 0.735 μA with GCE sensor to 33.3 and 11.1 μA with MWCNTs-CeO_2_@GCE and MWCNTs-Yb_2_O_3_@GCE sensors, respectively. This indicates that the sensitivity markedly increased with the modification of the electrode with MWCNTs-REMO films. The higher current peak at MWCNTs-CeO_2_@GCE compared to MWCNTs-Yb_2_O_3_@GCE and bare GCE confirms the better electrocatalytic effect of the CeO_2_ toward the anodic oxidation of SFX.

**Figure 8:**
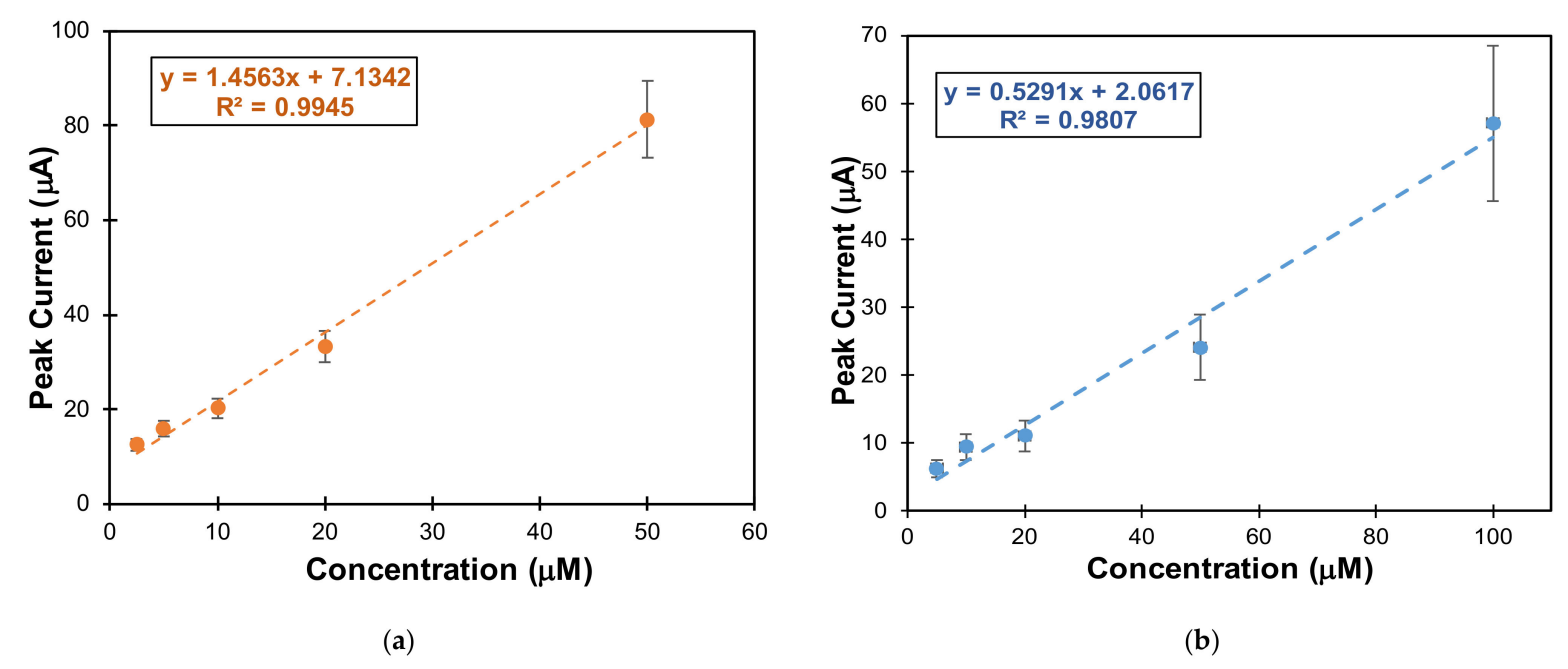
Calibration curves of SFX solutions in phosphate buffer solution, pH = 7.00 at 50 mV·s^−1^ using (**a**) MWCNTs-CeO_2_@GCE, and (**b**) MWCNTs-Yb_2_O_3_@GCE sensors. Experimental conditions: counter electrode: Pt, reference electrode: Ag/AgCl; room temperature; stagnant solution, modified GCE sensor: 2 mg/mL MWCNTs and 0.5 mg/mL CeO_2_ or Yb_2_O_3_ and 10 μL/mL of 5% Nafion solution.

The calibration curves of SFX in phosphate buffer (pH = 7.0) with MWCNTs-CeO_2_@GCE sensor in the concentration range 1.0–50.0 μM and MWCNTs-Yb_2_O_3_@GCE sensor in the concentration range 5.0–100.0 μM are plotted in Figure 8.

Linear graphs were obtained for both modified GCE sensors with better sensitivity using MWCNT-CeO_2_@GCE sensor (R^2^ = 0.9945). These results demonstrate that MWCNTs-CeO_2_@GCE and MWCNTs-Yb_2_O_3_@GCE sensors have better sensitivity in comparison to the bare GCE and can detect lower SFX concentrations in water (phosphate buffer at pH = 7.0). Furthermore, MWCNTs-CeO_2_@GCE showed the highest sensitivity and the lowest detection limit. The estimated limit of detection (LoD) based on standard deviation of repeated CVs of 5 μM SFX aqueous solution in phosphate buffer pH = 7.0 using MWCNTs-CeO_2_@GCE and MWCNTs-Yb_2_O_3_@GCE sensors, were 0.4 and 0.7 μM.

**Figure 9:**
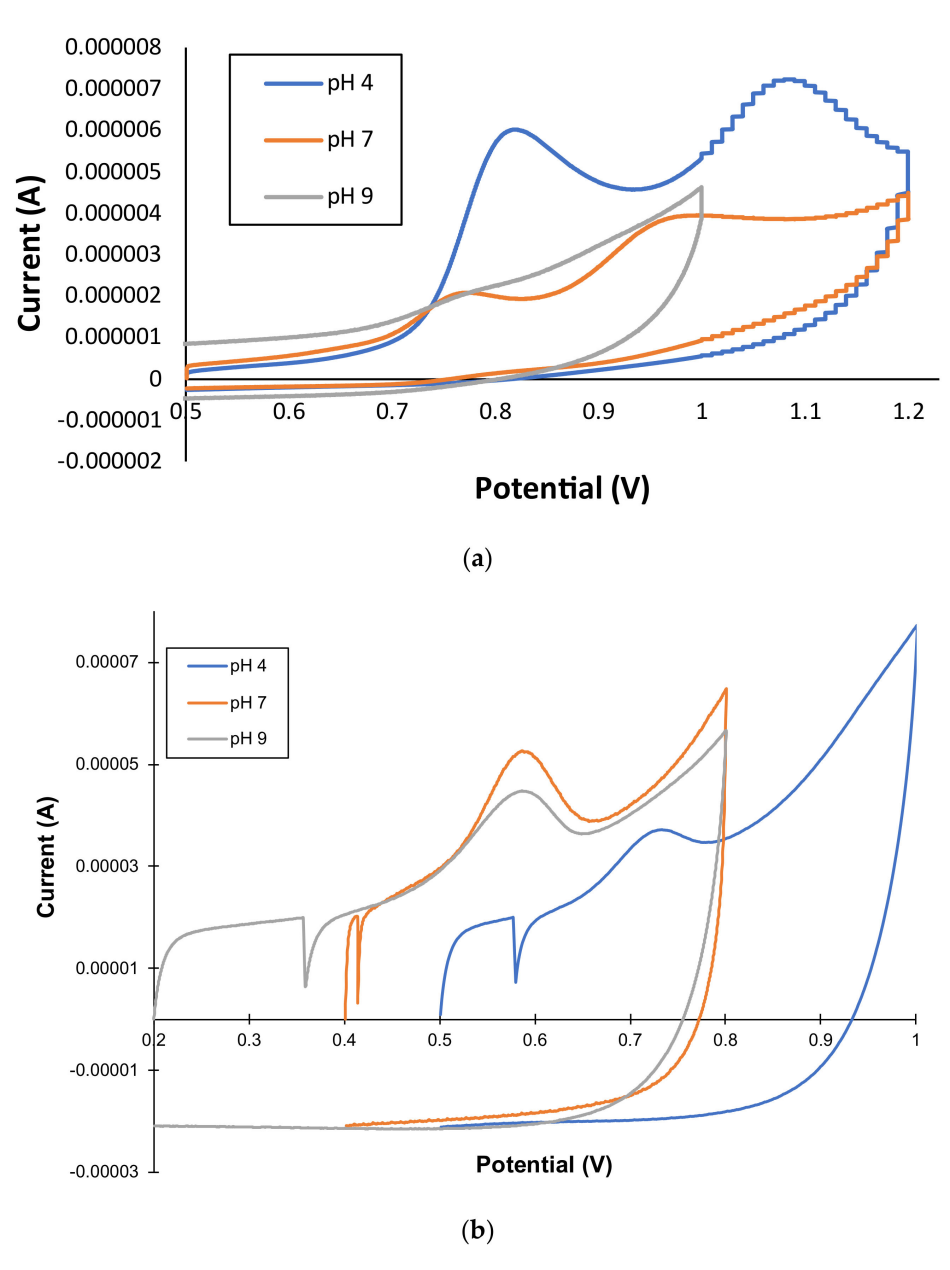

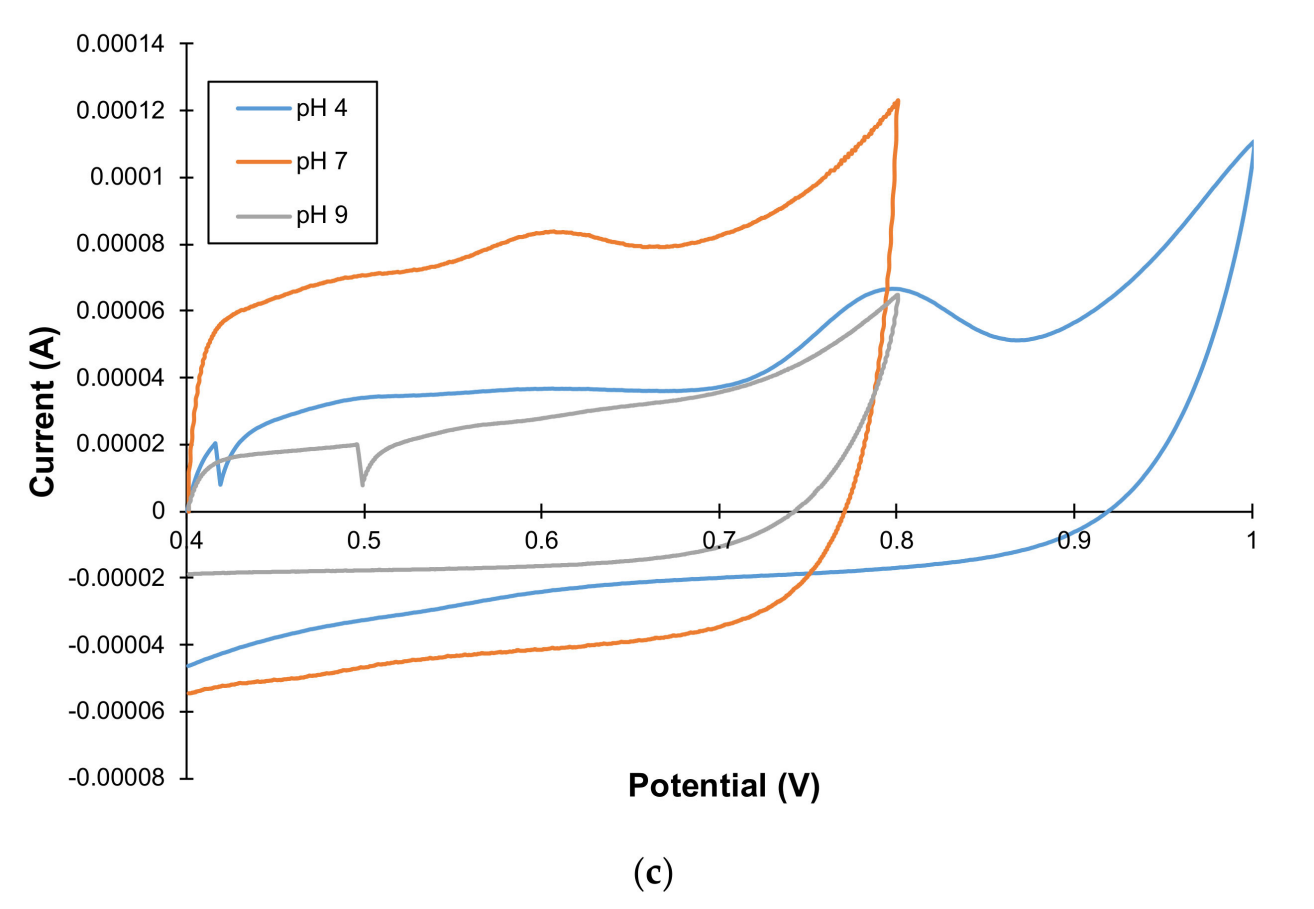
Anodic cyclic voltammograms of 100 μM SFX using (**a**) bare GCE, (**b**) MWCNTs-CeO_2_@GCE, and (**c**) MWCNTs-Yb_2_O_3_@GCE sensors in different buffer aqueous solutions (acetate: pH = 4.0, phosphate: pH = 7.0, and borate: pH = 9.0) at 50 mV·s^−1^. Experimental conditions: counter electrode: Pt, reference electrode: Ag/AgCl; room temperature; stagnant solution; modified GCE sensor: 2 mg/mL MWCNTs and 0.5 mg/mL CeO_2_ or Yb_2_O_3_ and 10 μL/mL of 5% Nafion solution.

Figure 9 presents the voltammograms of SFX using bare GCE and modified GCE sensors in different buffer solutions (acetate: pH = 4.0, phosphate: pH = 7.0, and borate: pH = 9.0) at 50 mV·s^−1^.

It is clear that the pH affects largely the electrochemical behavior of SFX using bare GCE and both MWCNTs-CeO_2_@GCE and MWCNTs-Yb_2_O_3_@GCE sensors. At pH = 9.0, SFX has the lowest electroactivity for the bare GCE and MWCNTs-CeO_2_@GCE and MWCNTs-Yb_2_O_3_@GCE sensors. The highest electroactivity of SFX was observed at pH = 4.0 using GCE (Figure 9a) and at pH = 7.0 for the two modified GCE electrodes (Figure 9b,c). This can be related to the acid-base properties of SFX that exists in the protonated form in acidic medium facilitating its adsorption on bare GCE sensors. However, at a neutral pH medium, its reactivity on MWCNTs-CeO_2_@GCE and MWCNTs-Yb_2_O_3_@GCE will be higher due the stronger intermolecular forces that can be formed through the non-bonding electron pairs on nitrogen atoms. This explains why it is recommended to use a phosphate buffer as a preferred medium for analysis of SFX.

The stability of the MWCNT-CeO_2_ and MWCNT-Yb_2_O_3_ was assessed by measuring the first peak current of a fixed concentration of SFX (5 µM) after the modified electrodes were stored for at room temperature and the peak current was measured every day for two weeks. A relative standard deviation (RSD) of 12% and 15% were measured for MWCNT-CeO_2_ and MWCNT-Yb_2_O_3_, respectively. The reproducibility of MWCNT-CeO_2_ and MWCNT-Yb_2_O_3_ electrodes was evaluated by using five independent electrodes and checking the current response of a 5 µM SFX solution. The RSD values calculated for both electrodes were less than 5%, indicating excellent reproducibility of the method. The precision of the method was evaluated by repeating five sequential measurements in the same solution containing 5 µM SFX using the same MWCNT-CeO_2_ and MWCNT-Yb_2_O_3_ electrodes. The RSD values calculated for both modified electrodes were found to be in the range of 10–15%, indicating that MWCNT-CeO_2_ and MWCNT-Yb_2_O_3_ are more suitable as disposable electrodes.

All in all, and as previously mentioned, the analysis of SFX have been reported in literature using different approaches [14,15,16,17,18,19,20,21]. All these methods have achieved low LoD and had acceptable sensitivity towards the drug; however, and as previously discussed they are time consuming and require several steps of extraction and concentration to analyze SFX samples. Recently, Karimi-Maleh et al. [21] have developed an electrochemical sensor based on CuO/SWCNTs and 1-butyl-3-methylimidazolium hexafluorophosphate for the analysis of SFX. This complex electrochemical sensor has achieved a LoD using SWV method. The results of CV analysis of SFX using sensors based on MWCNTs-REMO modified GCE showed excellent sensitivity and detected low concentrations of SFX ≥ 0.4 μM.

## 4. Conclusions

In this work, new electrochemical sensors based on the modification of GCE surface with MWCNTs-CeO_2_ and MWCNTs-Yb_2_O_3_ for the analysis of SFX in water samples was developed. REMO nanoparticles were manufactured using the hydrothermal method in presence and absence of the anionic surfactant, Triton^TM^ X-100. Hybrids of MWCNTs and REMO were utilized to modify the GCE. The MWCNTs-CeO_2_ and MWCNTs-Yb_2_O_3_ exhibited very high sensitivity and detected low concentrations of SFX as a representative of sulfonamides. The detection limit of the newly developed MWCNTs-REMO@GCE sensors was in the range of 0.4–0.7 μM for SFX. The successful results obtained with MWCNTs-CeO_2_@GCE and MWCNTs-Yb_2_O_3_@GCE sensors confirm that these electrochemical sensors have high sensitivity. The CV method using MWCNTs-REMO@GCE sensors have a great potential to be applied to analyze other sulfa drugs in real water, food, and biological samples. Due to the simple experimental procedures, short analysis time, high sensitivity and possible implementation in a portable device format, the developed method based on MWCNTs-REMO@GCE sensors are promising devices to analyze drugs in water and biological samples within a very short time.

## Data Availability

The data presented in this study are available within this article. Further inquiries could be directed to the authors.
